# Tightly Coupled Integration of Ionosphere-Constrained Precise Point Positioning and Inertial Navigation Systems

**DOI:** 10.3390/s150305783

**Published:** 2015-03-10

**Authors:** Zhouzheng Gao, Hongping Zhang, Maorong Ge, Xiaoji Niu, Wenbin Shen, Jens Wickert, Harald Schuh

**Affiliations:** 1 School of Geodesy and Geomatics, Wuhan University, 129 Luoyu Road, Wuhan 430079, China; E-Mails: zhouzhenggao@126.com (Z.G.); wbshen@sgg.whu.edu.cn (W.S.); 2 GNSS Research Center, Wuhan University, 129 Luoyu Road, Wuhan 430079, China; E-Mail: xjniu@whu.edu.cn; 3 German Research Centre for Geosciences (GFZ), Telegrafenberg, Potsdam 14473, Germany; E-Mails: maorong.ge@gfz-potsdam.de (M.G.); wickert@gfz-potsdam.de (J.W.); harald.schuh@gfz-potsdam.de (H.S.)

**Keywords:** global navigation satellite system (GNSS), inertial navigation system (INS), GNSS/INS tightly coupled integration, ionospheric-constrained precise point positioning, re-convergence

## Abstract

The continuity and reliability of precise GNSS positioning can be seriously limited by severe user observation environments. The Inertial Navigation System (INS) can overcome such drawbacks, but its performance is clearly restricted by INS sensor errors over time. Accordingly, the tightly coupled integration of GPS and INS can overcome the disadvantages of each individual system and together form a new navigation system with a higher accuracy, reliability and availability. Recently, ionosphere-constrained (IC) precise point positioning (PPP) utilizing raw GPS observations was proven able to improve both the convergence and positioning accuracy of the conventional PPP using ionosphere-free combined observations (LC-PPP). In this paper, a new mode of tightly coupled integration, in which the IC-PPP instead of LC-PPP is employed, is implemented to further improve the performance of the coupled system. We present the detailed mathematical model and the related algorithm of the new integration of IC-PPP and INS. To evaluate the performance of the new tightly coupled integration, data of both airborne and vehicle experiments with a geodetic GPS receiver and tactical grade inertial measurement unit are processed and the results are analyzed. The statistics show that the new approach can further improve the positioning accuracy compared with both IC-PPP and the tightly coupled integration of the conventional PPP and INS.

## 1. Introduction

Nowadays, precise point positioning (PPP) [1] is considered a very efficient approach for real-time precise positioning services and is widely used in static and kinematic positioning [2,3,4,5]. However, GPS needs usually at least four or more observed satellites to provide precise positions. Unfortunately, this condition cannot always be satisfied, especially if a vehicle is going through an urban canyon or tunnel or under a forest canopy. Therefore, reliable positioning using GPS alone in difficult urban situations is still a serious challenge. Furthermore, the long convergence at the beginning and after a serious loss of lock on satellites, which can happen frequently in kinematic applications in the above-mentioned severe observation situations is still a major weakness of PPP, although the reconvergence can be shortened by properly estimating carrier phase cycle slips over the data gaps [6,7].

In contrast with GPS, the Inertial Navigation System (INS) can provide continuous positioning information by processing Inertial Measurement Unit (IMU) measurements without any external aids after the required initialization and alignment [8]. However, the positioning accuracy of INS degrades rapidly over time due to IMU sensor errors, especially for low cost IMUs [9]. Such IMU sensor errors can easily be parameterized, but can only be precisely estimated and compensated using external information, such as GPS observations or known positions [10]. 

Hence, interest in exploring the integration of GPS and INS has been growing for decades. In most of the research on GNSS/INS integrations for precise positioning applications, differential GPS positioning is involved in the loose or tightly coupled integration for its high accuracy character [11,12,13,14]. However, its requirement of nearby reference stations restricts its applications for platforms moving over large areas, such as in mobile mapping systems and aerial surveying. Thanks to the rapid development in the real-time PPP, related services are now available any time and everywhere with a single receiver. Recently, PPP using ionosphere-free combination (LC-PPP) was introduced into tightly coupled integration systems with INS [15,16,17]). LC-PPP/INS tightly coupled integration has been a hot topic for many years. The related studies show the integration can not only overcome the weaknesses in each individual system, but also improve its accuracy, reliability, and continuity. Even when the number of GPS satellites is not enough for PPP computation, these observations can still contribute to the tightly coupled integration to minimize the diffuse trend of INS errors. According to the study by Zhang and Gao [15], the tightly coupled integration between conventional PPP and tactical grade IMU can provide vehicle positions with an accuracy of about 10 cm and 15 cm in the horizontal and vertical, respectively. The research by Roesler and Martell [16] indicates a position accuracy in terms of Root Mean Square (RMS) of 15 cm in both the horizontal and vertical directions can be obtained by the tightly coupled integration between LC-PPP and INS based on the analysis of several airborne data sets using various IMUs of both tactical and navigation grades.

Besides the improvement in positions, the result also shows the data breaks can be easily recovered and carrier-phase ambiguities could be rapidly reinitialized with the help of INS for short GPS outages. Shin and Scherzinger [18] showed there would be no clear performance degradation caused by GPS outages within 10 s, while Du [19] claimed that INS can aid the cycle slip detection in tightly coupled LC-PPP/INS integration mode.

Recently, Li *et al* demonstrated that PPP using uncombined GPS observations with ionospheric constraints (IC-PPP) can improve the performance in terms of both accuracy and convergence [20,21,22]. In LC-PPP the carrier-phase and pseudo-range ionospheric delays are eliminated independently and the combined observation is three times as noisy as the raw ones. These may be the reason for the better performance of IC-PPP [21,22]. 

In this paper, a new strategy for the tightly coupled integration of GPS and INS is developed, whereby IC-PPP is employed instead of LC-PPP to further improve the accuracy and convergence of the integrated system. The mathematical model and algorithm of the new strategy is presented in detail. The related software package for validation of the algorithm is developed. In the evaluation of this tightly integration strategy, one airborne data set and one vehicle-borne data set of a geodetic GPS receiver and tactical INS are processed and analyzed. Besides, data with simulated and actual loss of lock on satellites are processed and the results are investigated in order to show the impact of tactical INS on the re-convergence of IC-PPP.

## 2. IC-PPP Observation Model

As is well known, the linearized observation equations of raw GPS pseudo-range and carrier-phase measurements can be expressed as [22]:

(1) $$L_{GPS,j}=\left|p_{r-}p^{s}\right|+u\;\delta p_{r}+c\left(t_{r}-t^{s}\right)-{\left({\text{λ}}_{j}/{\text{λ}}_{1}\right)}^{2}{\text{ρ}}_{ion,1}+{\text{ρ}}_{trop}-{\text{λ}}_{j}N_{j}+\text{Δρ}+{\text{ρ}}_{pw,j}+{\text{ε}}_{L,j}$$

(2) $$P_{GPS,j}=\left|p_{r-}p^{s}\right|+u\;\delta p_{r}+c\left(t_{r}-t^{s}\right)+{\left({\text{λ}}_{j}/{\text{λ}}_{1}\right)}^{2}{\text{ρ}}_{ion,1}+{\text{ρ}}_{trop}-{\text{γ}}_{j}\left({\text{ρ}}_{dcb}^{s}-{\text{ρ}}_{r,dcb}\right)+\text{Δρ}+{\text{ε}}_{P,j}$$

where, *j* (*j* = 1, 2) is the frequency of the observations with corresponding wavelength ${\text{ λ}}_{j}$, *P* and *L* are GPS pseudo-range and carrier-phase observations in length units, $p_{r}\;$ and $p^{s}$ are the user and satellite position vector in Earth Centered Earth Fixed (*e*)-frame, $\delta p_{r}$ and u are the position correction vector and the relative direction cosine vector of each satellite-receiver pair, $t_{r}$ and $\;t^{s}$ represent receiver and satellite clock offset, respectively, *c* is the speed of light in vacuum, ${\text{ρ}}_{ion,1}$ and ${\text{ρ}}_{trop}$ are ionosphere delay of *P*1 and troposphere delays, ${\text{ρ}}_{dcb}^{s}$ and ${\text{ρ}}_{r,dcb}$ are the differential code bias (DCB) of satellite and receiver, γ is the DCB coefficient for GPS pseudo-range (${\text{γ}}_{1}=f_{2}^{2}/(f_{1}^{2}-f_{2}^{2})$, ${\text{γ}}_{2}=f_{1}^{2}/(f_{1}^{2}-f_{2}^{2})$, *N* represents carrier-phase ambiguity, Δρ stands for the other corrections, such as relativity effects, earth rotation effects, antenna phase center offset and antenna phase center variation, ${\text{ρ}}_{pw}$ is carrier phase wind-up effect, ${\text{ε}}_{P}$ and ${\text{ε}}_{L}$ are the corresponding observation noises. 

As shown in Equation (1), ambiguities of *L*1 and *L*2 are estimated as parameters in the IC-PPP [20,21,22], which is different from the ionospheric free combination ambiguity used in LC-PPP. In this paper, the float ambiguities of IC-PPP and LC-PPP are used in PPP and PPP/INS parameter estimation. 

According to the study by Crespillo *et al* [23], the Doppler observation is important to minimize the effect of IMU sensor errors. Hence, they are also considered using the following observable equation:

(3) $$D_{GPS,j}{\text{λ}}_{j}=\left|v^{s}-v_{r}\right|+u\;\delta v_{r}+c\left({\dot{t}}_{r}-{\dot{t}}^{s}\right)-{\dot{\text{ρ}}}_{ion,1}+{\dot{\text{ρ}}}_{trop}+\text{Δ}\dot{\text{ρ}}+\varepsilon_{D,j}$$

where, $v^{s}$ and $v_{r}$ are the velocity vector of satellite and user in *e*-frame, $\delta v_{r}$is the user velocity corrections vector, ${\dot{t}}_{r}$ and ${\dot{t}}^{s}$ represent receiver clock drift and satellite clock drift, ${\dot{\text{ρ}}}_{ion,1}$ and ${\dot{\text{ρ}}}_{trop}$ are the variation of ionosphere delay and troposphere delay, $\text{Δ}\dot{\text{ρ}}$ denotes the variations of other errors, such as relativity effects and the Earth rotation effect, ${\text{ ε}}_{D,k}$is the Doppler observation noise. 

In IC-PPP, as shown in Equations (1) and (2), the ionosphere delay along the line of sight (LOS) on frequency *L1* for each satellite-receiver pair is estimated as an unknown parameter [20,21,22]. Since there are a large number of such ionospheric delay parameters, the solution is rather weak, for example ionosphere delay parameters and receiver DCBs are highly correlated [22]. Therefore, *a priori* ionosphere information and/or receiver DCB are needed in order to enhance the solution. 

In this paper, both the temporal constraint and the spatial constraint of ionospheric delay are considered. IGS Global Ionosphere Map (GIM) data are used to constrain the slant delay parameters of each satellite-receiver pair. The slant ionospheric delay can be parameterized by a random walk process in the following discrete form:

(4) $$\begin{array}{c} {\text{ρ}}_{ion,k}={\text{ρ}}_{ion,k-1}+{\text{ω}}_{k}\;\\ \;E\left({\text{ω}}_{k}\right)=0,D\left({\text{ω}}_{k}\right)=q_{ion}^{2}dt \end{array}$$

where $q_{ion}^{2}$ is the power spectral density of the dynamic noise. As the noise is elevation dependent for slant delays, the empirical power density is selected as [22]:

(5) $$q_{ion}^{2}=\{\begin{array}{c} {\text{σ}}_{ion,t}^{2}\;E\geq 30^{{}^{\circ}}\\ {\text{σ}}_{ion,t}^{2}/{\left(2\sin\left(E\right)\right)}^{2}\;E<30^{{}^{\circ}} \end{array}$$

We select 0.03 m/$\sqrt{h}$ as the empirical value for ${\text{σ}}_{ion,t}$ from our tests.

The slant ionospheric delay ${\text{ρ}}_{GIM,k}$computed from GIM can be expressed by the related parameter and a white noise as:

(6) $$\begin{array}{c} {\text{ρ}}_{ion,k}={\text{ρ}}_{GIM,k}+{\text{ω}}_{GIM,k}\;\\ \;E\left({\text{ω}}_{GIM,k}\right)=0,D\left({\text{ω}}_{GIM,k}\right)={\text{σ}}_{GIM}^{2} \end{array}$$

According to ionosphere temporal and spatial characteristics, the a priori variance of GIM data (${\text{σ}}_{ion}$) can be expressed as [21,22]:

(7) $${\text{σ}}_{GIM}^{2}=\{\begin{array}{c} {}[{\text{σ}}_{ion,0}^{2}+{\text{σ}}_{ion,1}^{2}\cos\left(B\right)\cos\left(\frac{t-14}{12}\pi \right)]/{\text{sin}}^{2}\left(E\right),\;otherwise\\ {\text{σ}}_{ion,0}^{2}/{\text{sin}}^{2}\left(E\right),\;t<8\;or\;20<t,or\;B>\pi /3 \end{array}$$

where, *E* is the satellite elevation, *B* is the latitude of the ionosphere pierce point (IPP), t is the corresponding local time at the IPP (0–24 h), ${\text{σ}}_{\text{ion},0}^{2}$ is the variance of the zenith delay, and ${\text{σ}}_{\text{ion},1}^{2}$ is the variance of ionosphere delay variation along latitude and local time. The ionosphere a priori variance is determined by Equation (7) with the empirical values ${\text{σ}}_{\text{ion},0}={\text{σ}}_{\text{ion},1}=0.3$m for GIM data. 

In the LC-PPP, the receiver DCB of LC pseudo-ranges is common to the observations of all satellites. Therefore, it can be absorbed by the receiver clock, so that no DCB parameter is estimated in LC-PPP. However, in IC-PPP using Equation (2), we have two receiver DCBs for *P*1 and *P*2 pseudo-ranges, respectively, and they could not be absorbed simultaneously by the single receiver clock parameter. Obviously, ignoring receiver DCBs leads to biased range observations. It is shown that estimating receiver DCB in IC-PPP can enhance the initial position accuracy and shorten the convergence [22]. Hence, the receiver DCB is estimated as random process as:

(8) $$DCB_{k}=DCB_{k-1}+{\text{ω}}_{k-1}$$

with:

(9) $$E\left({\text{ω}}_{k-1}\right)=0,D\left({\text{ω}}_{k-1}\right)=q_{DCB}^{2}dt$$

where, $q_{DCB}$ is the dynamic noise of receiver DCB with a default value of 0.03 m/$\sqrt{h}$.

It should be pointed out that the difference between the reference point of GPS and INS observations must be considered in the tightly coupled IC-PPP/INS integration. Usually the position and velocity in Equations (1)–(3) are related to that of INS by the following equations:

(10) $$\begin{array}{c} p_{r}=p_{INS}^{n}+L_{p,r}^{n}\;\\ v_{r}=v_{INS}^{n}+L_{v,r}^{n}\; \end{array}\}$$

where, $p_{INS}^{n}\;$and $v_{INS}^{n}$ are the position and velocity calculated by INS mechanization in navigation (*n*)-frame (North-East-Down) in IC-PPP/INS tightly coupled integration, $L_{p,r}^{n}$ and $L_{v,r}^{n}$ are the lever-arm correction for position and velocity [10]. 

In addition, the satellite elevation-dependent weight [24] is adopted for the *a priori* variance for GPS observations:

(11) $${\text{ σ}}^{2}=\{\begin{array}{c} {\text{σ}}_{0}^{2}\;E\geq \pi /6\\ {\left(0.5{\text{σ}}_{0}/\text{sin}(E)\right)}^{2},\;else \end{array}$$

The *a priori* variance for pseudo-ranges (*P*1, *P*2), carrier-phases (*L*1, *L*2) and Doppler (*D*) are 0.2 m, 0.002 m and 0.1 m/s, respectively.

## 3. INS Model

Usually, INS has a sampling rate of few hundreds of Hz, which is much higher than that of GPS. In the IC-PPP/INS tightly coupled integration, GPS and INS observations are updated in a different manner, although theoretically they could be processed in a unique way using the standard Kalman filter equations. Nowadays, in the integrated processing, the observation update is only carried out for GPS observations to obtain estimates and variance-covariance matrix of the state vector, while INS observations are used as known values in the time update after the removal of IMU sensor errors. Furthermore, the latter calculation is realized with the algorithm widely used in INS processing [25] instead of the standard time update. The following gives details of the INS update algorithm.

### 3.1. INS Observational Equations 

The observations of the tactical IMUs used in this study are the velocities increment ($\text{Δ}{\tilde{v}}^{b}$) and angles increment ($\text{Δ}{\tilde{\text{θ}}}^{b}$) in the body (*b*)-frame (Forward-Right-Down). They are obtained by integrating the specific force (${\tilde{f}}^{b}$) of the accelerometer (a) and the angular rate (${\tilde{\omega }}_{ib}^{b}$) of the gyroscope (g), respectively. The systematic errors [26] in these observations can be described simply by a linear function with a bias (b) and a scale factor (S) in this paper [10]:

(12) $$\begin{array}{c} \text{Δ}{\tilde{v}}^{b}=\left(I+S_{a}\right)\text{Δ}v^{b}+b_{a}\text{Δ}t=\int^{\text{​}}{\tilde{f}}^{b}dt\;\\ \text{Δ}{\tilde{\text{θ}}}^{b}=\left(I+S_{g}\right)\text{Δ}{\text{θ}}^{b}+b_{g}\text{Δ}t=\int^{\text{​}}{\tilde{\text{ω}}}_{ib}^{b}dt \end{array}\}$$

where $\text{Δ}v^{b}$ and $\text{Δ}{\text{θ}}^{b}$ are the unbiased velocities and attitudes increment in b-frame, I is the unit matrix. Usually, the bias and scale factor are further modelled by a first-order Gauss-Markov process with the following discrete model:

(13) $$\begin{array}{c} x_{k}=x_{k-1}\exp\left(-\text{Δ}t_{k}/T\right)+{\text{ω}}_{k-1}\;\\ E\left({\text{ω}}_{k-1}\right)=0,\;D\left({\text{ω}}_{k-1}\right)=q\text{Δ}t_{k}=2{\text{σ}}^{2}\text{Δ}t_{k}/T \end{array}\}$$

where, T is the correlation time, q is the power spectral density (PSD) of driving white noise (ω), σ is the variance of ω depending on the instability of IMU bias and scale factor.

### 3.2. INS Update

Assume that we have the estimates of all the bias and scale factor parameters in Equation (12), then the IMU sensor errors in observations could be compensated to obtain the so called “clear” IMU data for the INS mechanization for updating position ($p_{INS}^{n}$), velocity ($v_{INS}^{n}$) and attitude (θ) [10,25].

The position, velocity, and attitude derived by INS mechanization are fully based on integration of INS observations. Because there are no redundant observations, they can be considered as both estimated and predicted values. Their transition matrix needed in the covariance propagation can be achieved by the perturbation of INS mechanization using the Psi-angle error model [10,27]. Considering the state equations for other parameters, such as INS sensor bias (b) and scale factor (S), the residual of wet zenith component of troposphere delay (*WZTD*) and ionospheric delays (${\text{ρ}}_{ion,1}$), ambiguities ($N_{1}$,$\;N_{2}$), receiver clock offset ($t_{r}$) and drift (${\dot{t}}_{r}$), and receiver DCB, we obtain the transition matrix $\Phi_{k,k-1}$ for all the state parameters, and the final form of the state parameter vector can be written as:

(14) $$X={\left[\text{δ}p,\text{δ}v,\text{δ}\text{θ},\text{δ}b_{g},\text{δ}S_{g},\text{δ}b_{a},\text{δ}S_{a},\text{δ}t_{r},\text{δ}{\dot{t}}_{r},\text{δ}WZTD,\text{δ}DCB,\text{δ}N_{1},\text{δ}N_{2},\text{δ}{\text{ρ}}_{ion,1}\right]}^{T}$$

where δ denotes correction, and the corresponding state equation system for Equation (14) can be written as:

(15) $$X_{k,k-1}=\Phi_{k,k-1}X_{k-1}+\varepsilon_{k}$$

where $\varepsilon_{k}$ is the noise of state equations with zero mean and co-variance matrix Q. The state equations of some parameters are already introduced above, while the dynamic noise of velocity, attitude and IMU sensor errors depend on the IMU grade (See Table 1). The dynamic noise models of the receiver clock offset and drift by Brown *et al.* [28] are utilized in this study, and can be written as:

(16) $$\begin{array}{c} \text{δ}t_{r}=\text{δ}{\dot{t}}_{r}dt\;\\ q_{\text{δ}t_{r}}=Kc^{2}h_{0}/2\;\\ q_{\text{δ}{\dot{t}}_{r}}=2K{\text{π}}^{2}c^{2}h_{2} \end{array}\}$$

where $q_{\text{δ}t_{r}}$ and $q_{\text{δ}{\dot{t}}_{r}}$ are the drive noise of receiver clock power spectral density (PSD); $h_{0}=2.0e^{-19}$ and $h_{2}=2e^{-20}$; *K* (*K* = 4) is experiential amplification factor. Besides, the variations of receiver clock offset and drift, as well as WZTD are treated as a random walk process, which can be defined as:

(17) $$x_{k}=x_{k-1}+{\text{ω}}_{k-1},E\left({\text{ω}}_{k-1}\right)=0,D\left({\text{ω}}_{k-1}\right)=q^{2}\text{Δ}t_{k}$$

Satellites’ ambiguities are modeled as random constant, which can defined as:

(18) $$x_{k}=x_{k-1\;}+{\text{ω}}_{k-1},E\left({\text{ω}}_{k-1}\right)=0,D\left({\text{ω}}_{k-1}\right)=0$$

From Equation (15), the variance-covariance of the INS updated state parameter is predicted by:

(19) $$P_{k,k-1}=\Phi_{k,k-1}P_{k-1}\Phi_{k,k-1}^{T}+Q_{k-1}$$

With the calculated state vector $X_{k,k-1}$ and its covariance matrix $P_{k,k-1}$, synchronized GPS observations at epoch *k* are used to update all state parameters, especially those parameters describing INS sensor errors.

## 4. Synchronized GPS Update

When GPS measurements synchronized with current INS epoch are available, the IC-PPP/INS tightly coupled integration algorithm is applied. The measurement model can be written as:

(20) $$Z_{k}=H_{k}X_{k}+\epsilon_{k}$$

In the equation, $Z_{k}$ is the measurement innovation vector achieved by making difference between INS predicted measurement and GPS raw observations:

(21) $$Z_{k}=\left[\begin{array}{c} \begin{array}{c} P_{INS,1}^{m}-P_{GPS,1}^{m}\\ P_{INS,2}^{m}-P_{GPS,2}^{m}\\ L_{INS,1}^{m}-L_{GPS,1}^{m} \end{array}\\ \begin{array}{c} L_{INS,2}^{m}-L_{GPS,2}^{m}\\ \begin{array}{c} D_{INS,1}^{m}-D_{GPS,1}^{m}\\ Ion_{INS}^{m}-Ion_{GIM}^{m} \end{array} \end{array} \end{array}\right]$$

Here, *m* is the number of available GPS satellites, subscript “*INS*” and “*GPS*” represent the INS predicted value and GPS observation, subscripts “1” and “2” stand for GPS frequency *L1* and *L2*, “*GIM*” denotes the ionospheric delays calculated by the GIM model. It is worth mentioning again that the lever-arm correction in Equation (10) must be taken into account when it forms the measurement innovation using Equations (1)–(3). According to reference [29], the lever-arm values also can be estimated along with other parameters in a Kalman filter with an estimation accuracy of about centimeter level in the horizontal direction and decimeter level in the vertical direction. However, in this paper, the lever-arm is measured accurately in *b*-frame before the experiments are started. Then, it can be transformed to *n*-frame by attitude information for position and velocity lever-arm corrections. H is the design matrix obtained directly from Equations (1)–(3), Equation (6), and Equation (10); ϵ is the observation noise with the *a priori* variance matrix $R_{k}$:

(22) $$R_{k}=diag\left\{R_{P1},R_{P2},R_{L1},R_{L2},R_{D},R_{Ion}\right\}$$

In this equation, the *a priori* variance of pseudo-range, carrier-phase, and Doppler is achieved from Equation (11), and the *a priori* ionospheric delay variance is obtained from Equation (7).

Then, the Kalman filter is employed for the parameter estimation of IC-PPP/INS. Usually, the implementation of the Kalman filter can be divided into “time update” and “measurement update” phases. The time update stage is carried out in each IMU interval as described before and results in the predicted state vector $X_{k,k-1}$ and its covariance matrix $P_{k,k-1}$, so that the measurement update can be undertaken by considering Equations (15)and (19):

(23) $$X_{k}=X_{k,k-1}+K_{k}\left(Z_{k}-H_{k}X_{k,k-1}\right)$$

(24) $$P_{k}=(I-K_{k})P_{k,k-1}{\left(I-K_{k}\right)}^{T}+K_{k}R_{k}K_{k}^{T}$$

with the gain matrix $K_{k}$ given by:

(25) $$K_{k}=P_{k,k-1}H_{k}^{T}\left(H_{k}P_{k,k-1}H_{k}^{T}+R_{k}\right)$$

**Figure 1:**
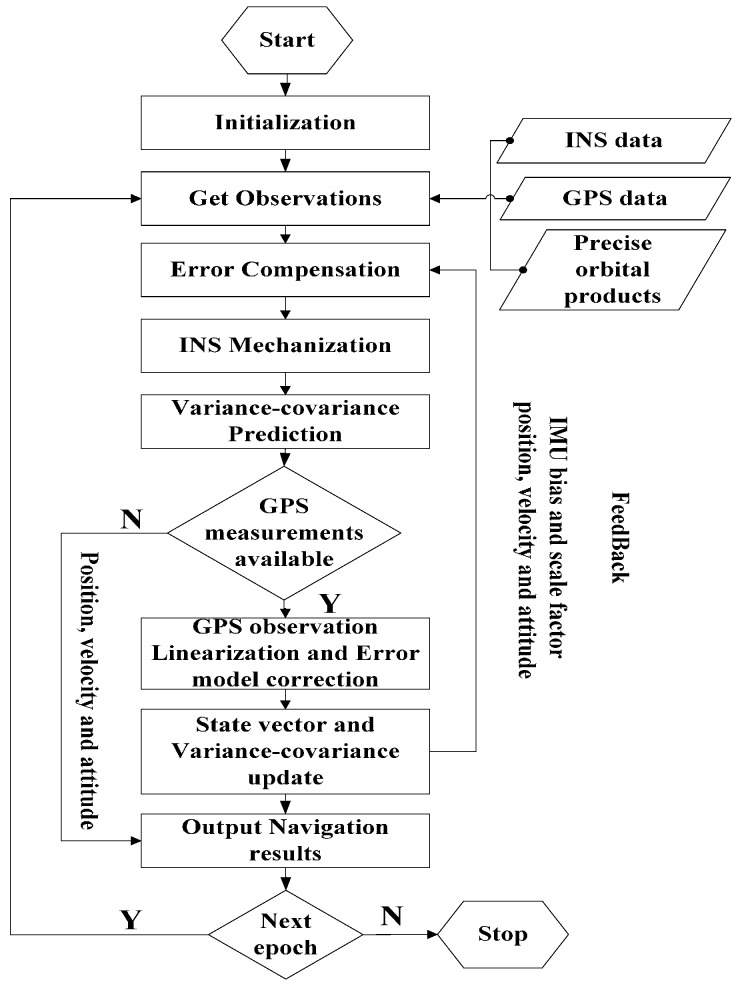
IC-PPP/INS tightly coupled integration algorithm.

The whole IC-PPP/INS tightly coupled integration processing procedure as described so far can be summarized in Figure 1. After the system initialization, the compensated IMU outputs in the form of velocity and angle increments are used in the INS mechanization. The INS update is carried out in every epoch no matter whether there are GPS measurements or not, and the related variance-covariance matrix is predicted by the Kalman filter in Equation (19). As soon as the synchronized GPS measurements are available, the Kalman “measurement update” is adopted for the update of state parameters and their variance-covariance matrix with Equations (23)–(25). Then, the corrected state parameters are obtained, and the IMU sensor errors will be the feedback for the IMU error compensation in coming epochs. 

## 5. Experimental Validation

**Figure 2:**
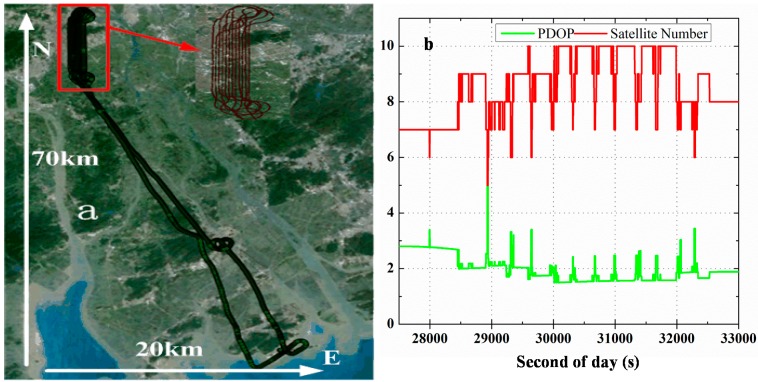
Trajectory of the airborne experiment (**a**) and the number of observed satellites and the PDOP (**b**) on 24 August 2014, in Guangzhou, China.

To evaluate the performance of IC-PPP/INS tightly coupled integration, two experiments were carried out. The first one was an airborne experiment performed on 24 August 2014 with a Trimble BD982 GNSS receiver and a POS310 (a tactical IMU from Wuhan MAP Space Time Navigation Technology Company), in Guangzhou, China. Shown in Figure 2a is the mission route with about 70 km in north-south direction and 20 km in east-west direction. The velocity of the plane is about ±45 m/s, ±50 m/s, and ±3 m/s in north, east, and vertical components, respectively. The second experiment was a vehicle-borne one performed with a NovAtel SPAN-FSAS in Wuhan, China, on 7 January 2012. The IMU used in SPAN-FASA is a tactical grade IMU from iMAR GmbH. Both the airborne and the vehicle-borne data covered about 1.6 hours. The sampling rate of the two tactical IMUs was 200 Hz and the sampling rate of GPS data was set to 1 Hz. The details of the two IMUs are shown in Table 1. 

**Table 1:** Technical Parameters of the POS310 and SPAN-FSAS units.

IMU		Dimensions and Weight	Operating Power and Temperature	Bias		Random Walk	
				Gyro. °/h	Acc. mGal	Angular °/s/$\sqrt{h}$	Velocity m/s/$\sqrt{h}$
**POS310**	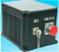	151 × 120 × 101 mm 2.2 kg	9~36 V, ≤15W −40 °C–60 °C	0.50	300	0.05	0.1
**SPAN-FSAS**	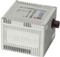	128 × 128 × 104 mm 2.1 kg	11~34 V, ≤16W −40 °C–71 °C	0.75	1000	0.03	0.1

The data were processed with three schemes: (1) IC-PPP; (2) IC-PPP/INS: tightly coupled integration of IC-PPP and INS; (3) LC-PPP/INS: tightly coupled integration of LC-PPP and INS. In the validation, the results are compared with that calculated using the Inertial Explorer (IE) software (NovAtel Company, Calgary, AB, Canada) as reference. As the fixed solution of GPS RTK calculated by GrafNav (according to Waypoint software technical reports, NovAtel Company) can provide 2–6 cm position accuracy when the baseline is less than 130 km in open sky [30] it is integrated with tactical grade INS and backward smoothing is carried out by the IE software, the result is in principle much better than that of the forward processing of the above-mentioned three schemes and thus serves as reference in this study. The accuracy of velocity and attitude of IE RTK integrated with tactical grade INS is about 1 cm/s and 0.02° [31]. In the preliminary process, we noticed that loss of lock on tracking satellites happens very frequently in the vehicle-borne experiment, therefore the related data processing is focused on the investigation of GPS signal outages.

### 5.1. Airborne Experiment

The trajectory of the airborne experiment are shown in Figure 2a, while the observed number of satellites and PDOP are plotted in Figure 2b. From our statistics, the average number of satellites is 8.1 and the mean PDOP 2.1. Obviously, the periodic change of number of satellites and the PDOP values (Figure 2b) are correlated with the route change (Figure 2a). The reason is that the bank angles for the plane to turn to the next mission route are about ±20 degrees, so that the satellites with low elevations may undergo loss of lock during the bank turns.

**Table 2:** RMS of the position, velocity, and attitude of the three processing schemes with respect to the reference solution using NovAtel IE software.

Process Modes	Position (cm)			Velocity (cm/s)			Attitude (°)		
	North	East	Down	North	East	Down	Roll	Pitch	Heading
IC-PPP	6.6	8.9	6.6	6.7	7.2	8.3	-	-	-
IC-PPP/INS	3.2	4.8	5.5	1.0	1.1	0.8	0.021	0.026	0.092
LC-PPP/INS	4.2	6.5	6.1	1.2	1.1	0.9	0.022	0.027	0.095

#### 5.1.1. Position, Velocity and Attitude Accuracy

**Figure 3:**
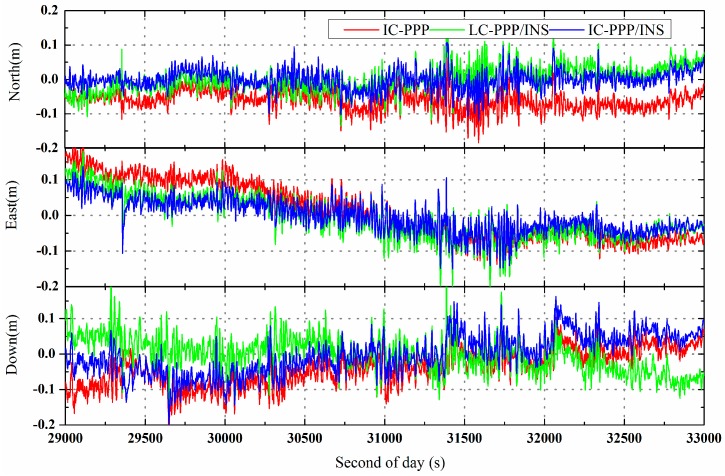
Position differences of IC-PPP (**red** line), LC-PPP/INS (**green** line), and IC-PPP/INS solution (**blue** line) with respect to the reference solution using NovAtel IE software.

The RMS of the differences in positions, velocities, and attitudes derived with IC-PPP, IC-PPP/INS, and LC-PPP/INS with respect to the reference solution are shown in Table 2. The time series of the position and velocity differences are shown in Figure 3.

According to the statistics of the airborne experiment, the position RMS in the north, east, and vertical directions are improved from 6.6 cm, 8.9 cm, and 6.6 cm of IC-PPP solution to 3.2 cm, 4.8 cm, and 5.5 cm of IC-PPP/INS solution, respectively. As expected, the position RMS of IC-PPP/INS is also smaller than that of LC-PPP/INS, with an improvement of about 1.0 cm, 1.7 cm, and 0.6 cm in the north, east, and vertical components, respectively. This is an obvious proof of the advantage of IC-PPP/INS compared with LC-PPP/INS. Besides, it is clear that both the position RMS of IC-PPP/INS and LC-PPP/INS are better than that of IC-PPP, confirming the necessity of the integration of GPS and INS. From Figure 3, there is a periodic variation in the north direction for all three solutions. This may correlate with the periodic change of the trajectory and the associated change of the observed satellites.

**Figure 4:**
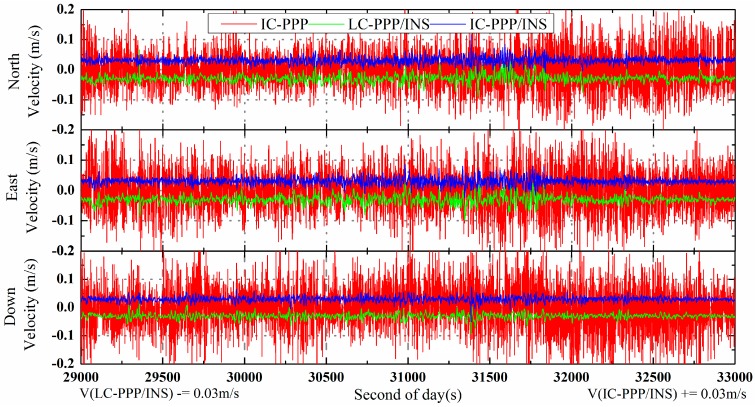
Velocity differences of IC-PPP (**red** line), LC-PPP/INS (**green** line), and IC-PPP/INS (**blue** line) with respect to the reference solutions; LC-PPP/INS and IC-PPP/INS velocities are shifted by −0.03 m/s and +0.03 m/s, respectively, for clarity.

For a better vision, the time series of velocity differences of LC-PPP/INC (green) is shifted by −3 cm/s and that of IC-PPP/INS +3 cm/s (blue) and is shown in Figure 4. From the statistics in Table 2, RMS of the IC-PPP solution is reduced by the IC-PPP/INS from 6.7 cm/s, 7.2 cm/s, and 8.3 cm/s to 1.0 cm/s, 1.1 cm/s, and 0.9 cm/s in the north, east, and vertical directions, respectively. The accuracy of LC-PPP/INS velocity is almost the same as that of IC-PPP/INS within 2 mm/s in terms of RMS, and the velocity accuracy of the vertical component is a little better than that of the horizontal components. 

**Figure 5:**
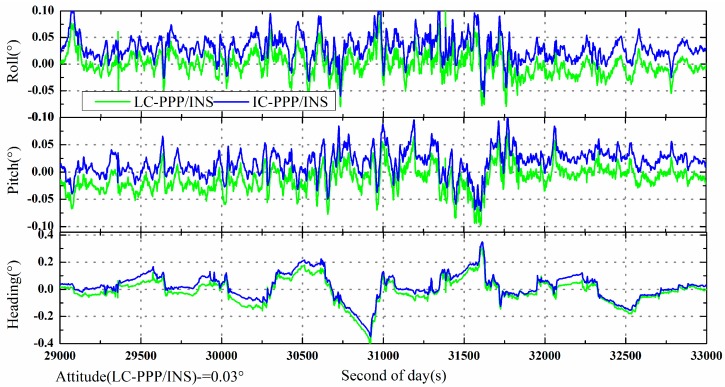
Attitude differences of the LC-PPP/INS and IC-PPP/INS (forward processing) with respect to the solution of IE software. The LC-PPP/INS differences are shifted by −0.03° for clarity.

The attitude differences of the IC-PPP/INS and LC-PPP/INC are shown in Figure 5. Like the velocities in Figure 4, the roll, pitch, and heading of LC-PPP/INS are shifted by −0.03°. According to the statistics in Table 2, the attitude RMS of both IC-PPP/INS and LC-PPP/INS are about 0.02°, 0.03°, and 0.09° for roll, pitch, and heading direction, respectively. Significantly, the accuracy of the heading component is worse than that of the roll and pitch, which is due to the poor observability in the heading direction compared with the other two directions. 

#### 5.1.2. Analysis of IMU Sensor Errors and PPP/INS Initialization 

**Figure 6:**
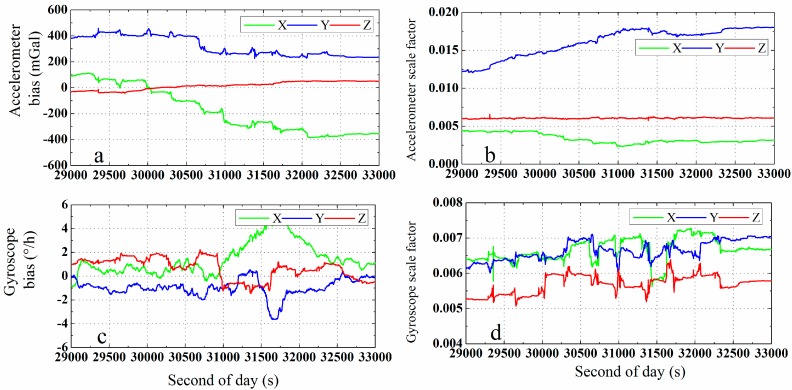
Estimated biases and scale factors of the POS310 IMU in the directions of the *b*-frame axes: accelerometer biases (**a**) and scale factors (**b**), gyroscope biases (**c**) and scale factors (**d**).

As mentioned above, IMU sensor errors can also be estimated and compensated online in the GPS/INS integration system. The estimated error parameters of the POS310 IMU sensor, *i.e.*, the bias and scale factor of the accelerometer and gyroscope, are shown in Figure 6a–d, respectively. According to the plots, the estimated IMU errors meet the IMU specifications in Table 1.

Besides, to show the position performance of tactical IMU in INS-only positioning mode, we did GPS outage simulations (no GPS observation were used) by adding an outage of 1, 5, 10, 15, 30 and 60 s, respectively, every 1000 s. The statistics of the position differences between the solutions with simulated outages and the reference are shown in Table 3. The averaged three-dimensional position offsets are smaller than 10 cm when the outage time is less than 5 s. The position accuracy could get worse up to about 56 cm, meter level, and even ten meters, if the outage time extends to 15, 30 and 60 s, respectively.

**Table 3:** Performance of INS-only positioning by using tactical IMU (POS310).

Position Offset	GPS Outage Time					
	1 s	5 s	10 s	15 s	30 s	60 s
**North (cm)**	0.8	6.5	23.4	56.3	266.9	976.8
**East (cm)**	0.9	5.7	13.6	23.2	91.3	522.8
**Down (cm)**	0.5	2.6	7.4	14.2	37.8	146.3

**Figure 7:**
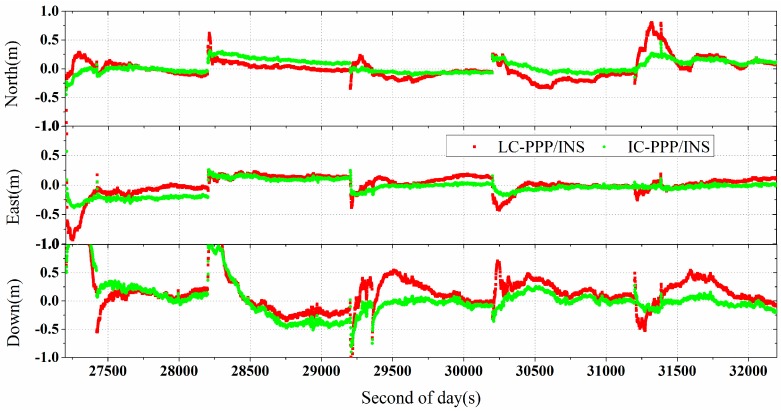
Position differences of LC-PPP/INS and IC-PPP/INS (**red** line) for PPP/INS (**green** line) initialization analysis.

To analyze the PPP/INS initialization performance, the airborne data was divided into five 1000 s parts. Each part was processed independently in both the LC-PPP/INS and IC-PPP/INS model. As the results shown in Figure 7 indicate, it is clear that the initialization performance of IC-PPP/INS is better than that of LC-PPP/INS in the horizontal component. IC-PPP/INS performs better than LC-PPP/INS in the vertical direction except in the second part.

### 5.2. Vehicle-Borne Experiment 

**Figure 8:**
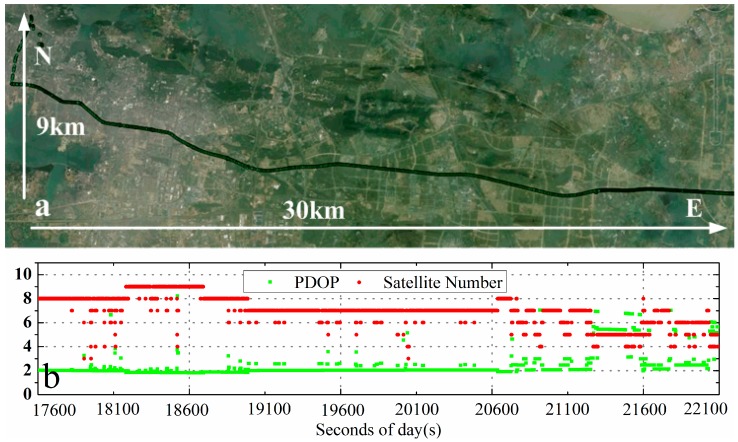
Trajectory of the vehicle-borne experiment (**a**) and number of satellites and PDOP (**b**) on 7 January 2012 in Wuhan, China.

The vehicle-borne experiment was carried out in Wuhan City where the tracking situation was spatially very different along the trajectory as shown in Figure 8a. In this test, the mission area was about 9 km long in north-south length and 30 km in the east-west direction (Figure 8a), and the velocity was about 0–±22 m/s. The number of tracked satellites and PDOP, with the average of 7.0 and 2.8, respectively, are shown in Figure 8b. From the number of tracked satellites, loss of lock on satellites happened rather frequently, especially in the second half. Anyway, to evaluate the IC-PPP/INS performance in urban environments, the data were processed in a similar way as the airborne experiments. Furthermore, the data with frequent GPS outages was also processed and investigated.

#### 5.2.1. Result of Data with Good GPS Continuity

**Figure 9:**
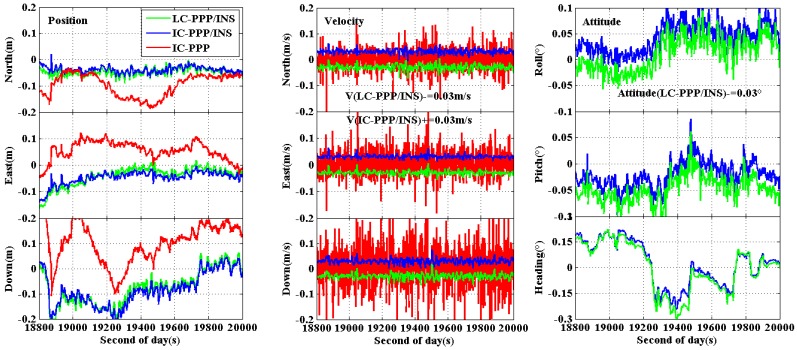
Differences of positions, velocities and attitudes of IC-PPP (**red** line), LC-PPP/INS (**green** line) and IC-PPP/INS (**blue** line) with respect to the reference solutions; LC-PPP/INS and IC-PPP/INS velocities are shifted by −0.03 m/s and +0.03 m/s, and the LC-PPP/INS attitudes are shifted by −0.03° for clarity.

Data over the period from 18,800 s to 20,000 s with a reasonable good GPS continuity was processed. Skipping the convergence period, RMS of the positions, velocities derived from IC-PPP, IC-PPP/INS, and LC-PPP/INS with respect to the reference solution are shown in Table 4 and the time series of the differences are shown in Figure 9. Similarly, for clarity the velocities of LC-PPP/INS, and IC-PPP/INS are shifted by −3 cm/s and +3 cm/s, respectively, and the LC-PPP/INS attitudes are shifted by −0.03°.

**Table 4:** RMS of the position, velocity, and attitude of the IC-PPP, IC-PPP/INS and LC-PPP/INS solutions of the vehicle-borne experiment.

Process Modes	Position (cm)			Velocity (cm/s)			Attitude (°)		
	North	East	Down	North	East	Down	Roll	Pitch	Heading
IC-PPP	10.6	6.5	12.7	3.6	4.1	7.4	-	-	-
IC-PPP/INS	4.0	5.3	10.7	0.8	0.6	0.9	0.048	0.030	0.141
LC-PPP/INS	4.9	5.5	10.6	0.9	0.7	0.9	0.052	0.030	0.146

From Table 4, the IC-PPP position is improved from 10.6 cm, 6.5 cm, and 12.7 cm to 4.0 cm, 5.3 cm, and 10.7 cm by IC-PPP/INS, respectively. The RMS of IC-PPP/INS is slightly better than that of LC-PPP/INS within 1 cm in the horizontal direction, but the RMS of IC-PPP/INS is about 0.1 cm worse than that of LC-PPP/INS in the vertical component. Similar to the comparison of the airborne experiments, the velocity accuracy of both IC-PPP and LC-PPP/INS is improved to several millimeters per second by IC-PPP/INS in the horizontal direction, but no improvement can be seen in the vertical component. The attitude accuracy of SPAN-FSAS in this vehicle test is about 0.05°, 0.03° and 0.14° in the roll, pitch, and heading components. From the RMS in Table 2 and Table 4, the vehicle-borne results are comparable with those of the airborne experiments when the GPS continuity is good.

#### 5.2.2. Result of Data with Frequent GPS Outages

**Figure 10:**
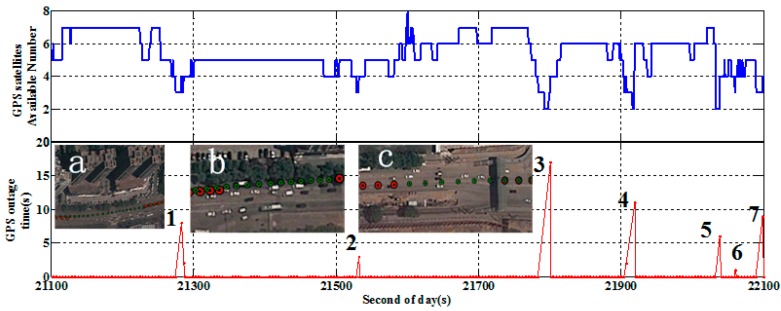
Vehicle-borne satellite availability (**top**) and the GPS outages situations (**bottom**, **a** shows urban canyon with high and big buildings; **b** is under foliage shade; **c** is under an overpass), on 7 January 2012 in Wuhan, China.

As shown in Figure 8, the GPS data quality of the second half time is rather poor due to a number of loss of lock on satellites events. There are seven severe GPS outages after the 21,100 s of day mark shown in Figure 10 with the outage length. Among all the outages, outages 1, 2, 3 and 6 are in an urban canyon with high and big buildings as shown in Figure 10a (red points are calculated by IC-PPP and green points are calculated by IC-PPP/INS); Outages 4 and 7 are under foliage shade (Figure 10b); Outage 5 is under an overpass (Figure 10c). From the data availability of each satellite shown on the same figure, these outages are related to a loss of satellite lock. As shown in the bottom figure of Figure 10 and Table 5, the maximum outage time is 17 s and the time between two adjacent outages it is less than 300 s. 

**Table 5:** The GPS outage time last and available GPS satellite number.

Outage Scenes	1	2	3	4	5	6	7
Outage time	8 s	3 s	17 s	11 s	6 s	1 s	9 s
Satellite number	3	3	2.6	2.7	2	3	3

Clearly, although there are some available GPS satellites (2–3 satellites) during the outages, these satellite observations are not enough for PPP computation. Therefore, these outages lead to an essential reinitialization and consequently the position accuracy is degraded seriously when using GPS only for positioning. However, the “not enough observations” can be used in the PPP/INS tightly coupled integration to assist the parameter estimation. Due to the INS’ character of high short-term accuracy, a more reliable and accurate positioning result can be obtained by using PPP/INS tightly coupled integration.

**Figure 11:**
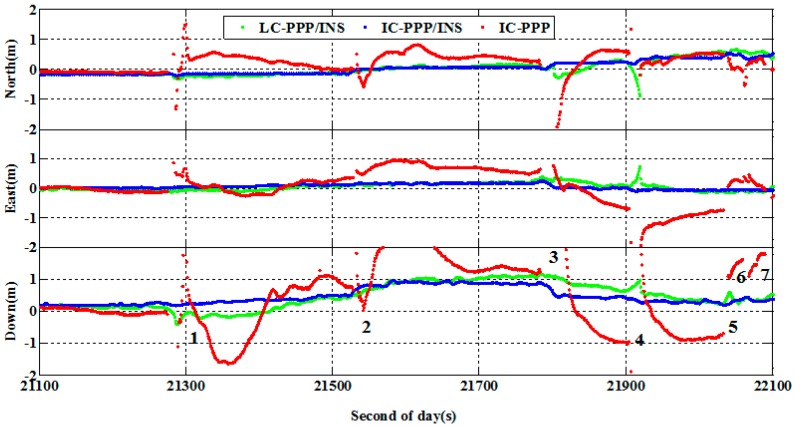
Position differences of IC-PPP (**red** line), LC-PPP/INS (**green** line) and IC-PPP/INS solution (**blue** line) for the data with frequent GPS outages, on 7 January 2012 in Wuhan, China.

To show the performance of IC-PPP/INS over the outage period, the data was processed in IC-PPP and IC-PPP/INS mode, respectively. The position differences of the IC-PPP, LC-PPP/INS and IC-PPP/INS solutions with respect to the reference solution are shown in Figure 11. Over the whole period there are reinitializations indicated by very large position differences. This is obviously because of a lack of sufficient GPS observations for the PPP calculation. However, in the cases of partial outages, the LC/IC-PPP/INS mode can still work well due to the INS contribution. Even if all GPS satellites are lost, INS mechanization can also provide navigation solutions based on IMU outputs alone. Hence, no matter whether a full or partial outage happens, tightly coupled IC-PPP/INS integration can provide the user with reasonable navigation results.

According to Figure 11, although the position accuracy of LC/IC-PPP/INS solution is not as good as that without GPS outages, it is significantly better than that of the IC-PPP solution. Furthermore, there are almost no obvious reconvergences over the GPS outages in the LC/IC-PPP/INS solution. The statistics show that the position RMS of the IC-PPP is improved by IC-PPP/INS from 45 cm, 56 cm, and 124 cm to 19 cm, 12 cm, and 41 cm with an improvement of 57%, 78%, and 67% in the north, east and vertical directions. Besides, the position RMS of IC-PPP/INS is also smaller than that of LC-PPP/INS, with an improvement of about 21%, 11%, and 23% in the north, east, and vertical components, respectively.

As shown in Table 3, in LC/IC-PPP/INS mode, it is possible to achieve reliable positioning solutions by INS mechanization during short GPS outages using tactical IMU sensors. Therefore, compared to IC-PPP, with the help of INS carrier-phase ambiguities can converge rapidly in LC/IC-PPP/INS mode after GPS outages [18], which can improve the position accuracy in the reinitialization process and reduce the reconvergence time. 

## 6. Conclusions

In this paper, IC-PPP instead of LC-PPP is utilized in a tightly coupled integration of GPS and INS. The mathematical model and associated algorithm of the new approach are implemented and validated. 

The performance of the IC-PPP/INS tightly coupled integration is investigated by analyzing data from an airborne and a vehicle-borne experiment. The results confirm that IC-PPP/INS could improve the performance of the LC-PPP/INS and IC-PPP in terms of position and velocity accuracy. From the airborne and vehicle-borne data without frequent GPS outages, IC-PPP/INS can provide positions and velocities of better than 10 cm and a few mm/s in terms of RMS. The attitude accuracy of IC-PPP/INS is about 0.02°–0.05° for roll and pitch, and 0.09°–0.14° for heading, a little better than that of LC-PPP/INS, with an improvement of 3%–7%.

Through the analysis and investigation of data with frequent GPS outages, we demonstrated that IC-PPP/INS and LC-PPP/INS can still provide positions with reasonable accuracy during certain GPS outages, and on average, IC-PPP/INS could improve the accuracy of IC-PPP by 57%, 78% and 67% and that of LC-PPP/INS by 21%, 11% and 23% in the north, east and vertical components, respectively. In other words, IC-PPP/INS can significantly improve the position accuracy during the reinitialization process and reduce the reconvergence time. In this paper IC-PPP/INS also shows a better initialization performance than that of LC-PPP/INS.
